# Assessment of a new technique for biological augmentation in sternoclavicular joint dislocations: a cadaveric feasibility and biomechanical evaluation

**DOI:** 10.1007/s00068-026-03194-5

**Published:** 2026-04-30

**Authors:** Ole Somberg, Eckart Förster, Thomas Rosteius, Maria Bernstorff, Thomas Schildhauer, Matthias Königshausen

**Affiliations:** 1 Department of General and Trauma Surgery, BG University Hospital Bergmannsheil, Buerkle-de-la-Camp- Platz 1, 44789 Bochum, Germany; 2 Institute of Anatomy, Ruhr-University, Universitätsstraße 150, 44801 Bochum, Germany

**Keywords:** Sternoclavicular joint (SCJ), Surgical technique, Periosteal flap, Stability, Cadaveric study

## Abstract

**Aims:**

Biological augmentation is necessary in chronic sternoclavicular dislocations. However, the placement of drill holes for a graft can be difficult at that region. The aim of this study was to evaluate, as an early proof-of-concept, the feasibility of using a locally available clavicular periosteal flap (CPF) or sternal periosteal flap (SPF) for sternoclavicular joint (SCJ) stabilization and to biomechanically test the periosteal flaps.

**Methods:**

A cadaveric anatomic feasibility and biomechanical study (level of evidence V) was performed on nine SCJs. The CPF was mobilized from three sides by lifting the flap off the bone, preserving its attachment near the SCJ. Two 1.6 mm drill holes were created in the sternum, and the CPF was folded over the SCJ and fixed with a non-resorbable wire. Additionally, the non-resorbable wire was used in terms of a figure-of-8-bracing through a 1.6 mm clavicular drill hole to secure the joint position. Flap dimensions and sternal overlap were measured. Vice versa SPFs were prepared in selected specimens and fixed in a similar fashion. The CPFs were biomechanically tested using a uniaxial tensile testing machine to obtain values for stress at 50% strain (σ (50%)), maximum force (Fmax), maximum stress (σmax), strain at maximum force (ε(Fmax)), and strain at failure (ε at failure).

**Results:**

CPF mobilization and fixation were successfully achieved in all specimens. The flaps showed an average length and width of 3.5 × 2.6 cm and a sternal overlap of 2.0 cm. In four specimens, sternal periosteal flaps (SPF) were additionally mobilized, with an average length and width of 3.0 × 2.6 cm and a clavicular overlap of 2.1 cm. Both flap types demonstrated adequate size for sufficient joint overlapping. Biomechanical testing of the CPFs yielded a mean stress at 50% strain (σ (50%)) of 0.30 ± 0.12 N/mm^2^, a maximum force (Fmax) of 18.55 ± 9.0 N, a maximum stress (σmax) of 0.48 ± 0.10 N/mm^2^, a strain at maximum force (ε(Fmax)) of 1.69 ± 0.82%, and a strain at failure (ε at failure) of 2.36 ± 1.41%.

**Conclusion:**

This cadaveric study provides an early proof-of-concept demonstrating the feasibility of a novel SCJ stabilization technique using a locally available CPF or SPF. While this approach may offer advantages by minimizing donor-site morbidity associated with graft harvesting and potentially reducing surgical complexity, further biomechanical and clinical studies are required to establish its effectiveness and clinical applicability.

**Level of evidence:**

V.

## Introduction

The sternoclavicular joint (SCJ) is a small yet crucial joint, as it represents the only bony connection between the shoulder girdle and the trunk. The ligamentous stabilization of the SCJ is provided by several key structures, including the anterior and posterior sternoclavicular ligaments, the costoclavicular ligament, and the interclavicular ligament. These ligaments work together to maintain joint integrity and allow controlled mobility. Among them, the posterior capsule plays the most critical role in limiting both anterior and posterior translation, while the anterior capsule significantly contributes to restraining anterior movement. In contrast, the costoclavicular and interclavicular ligaments have minimal influence on translational stability [1, 2]. SCJ dislocations are rare, representing approximately 3% of all shoulder injuries and less than 1% of all joint dislocations [3]. SCJ dislocations can be classified according to several criteria. One way to categorize them is based on the time elapsed since the injury, distinguishing between acute and chronic dislocations. Another classification is based on the direction of the dislocation, differentiating between anterior and posterior dislocations. Additionally, SCJ dislocations can be categorized by their underlying cause as either traumatic, typically resulting from high-energy impact or contact sports, or atraumatic, which may be related to ligamentous laxity or connective tissue disorders. The most common cause is indirect trauma, such as a fall onto the shoulder, which typically results in an anterior dislocation of the clavicle, while posterior or retrosternal dislocations are usually caused by a direct blow to the front of the clavicle [4]. Additionally, apart from traumatic mechanisms, hyperlaxity can predispose individuals to atraumatic dislocations of the sternoclavicular joint. Anterior dislocations are significantly more frequent than posterior ones, with an estimated occurrence ratio of 20:1 [5, 6]. 

Acute anterior SCJ dislocations are often managed conservatively after successful reduction, whereas acute posterior dislocations require urgent reduction due to the risk of mediastinal injuries [7]. In cases of chronic SCJ instability or dislocation, a combination of suture augmentation and tendon grafts is frequently employed to achieve joint stabilization [8–10]. Increasingly, authors emphasize the potential benefits of this strategy even in the acute setting of anterior and posterior dislocations, suggesting that biological augmentation using suture repair and tendon autografts or anterior capsule repair may be advantageous already at an early stage [11–14]. The aim of this cadaver study was to assess, as an early proof-of-concept, whether a clavicular periosteal flap (CPF) or sternal periosteal flap (SPF) could be mobilized to explore the feasibility of a novel technique. We hypothesized that local CPFs and SPFs would provide sufficient dimensions and basic tensile strength to serve as a biological augmentation for SCJ reconstruction. This approach could potentially reduce the diameter of required sternal or clavicular drill holes, utilize a locally available flap, and thereby obviate the need for hamstring graft harvesting.

## Methods

### Demographic data

In our cadaveric anatomic feasibility and biomechanical study, we used *n* = 5 cadavers (*n* = 9 SCJs available in 2 female, 3 male) with an average age of 79 ± 3.4 years. No other pre-existing conditions of the body donors were known. On inspection, there was no evidence of prior surgeries involving the SCJ. The body donors derive from the donor program of Ruhr-University Bochum. Ethical approval was obtained from the local Ethics Committee (2024-591-f-S).

### Surgical technique

**Fig. 1:**
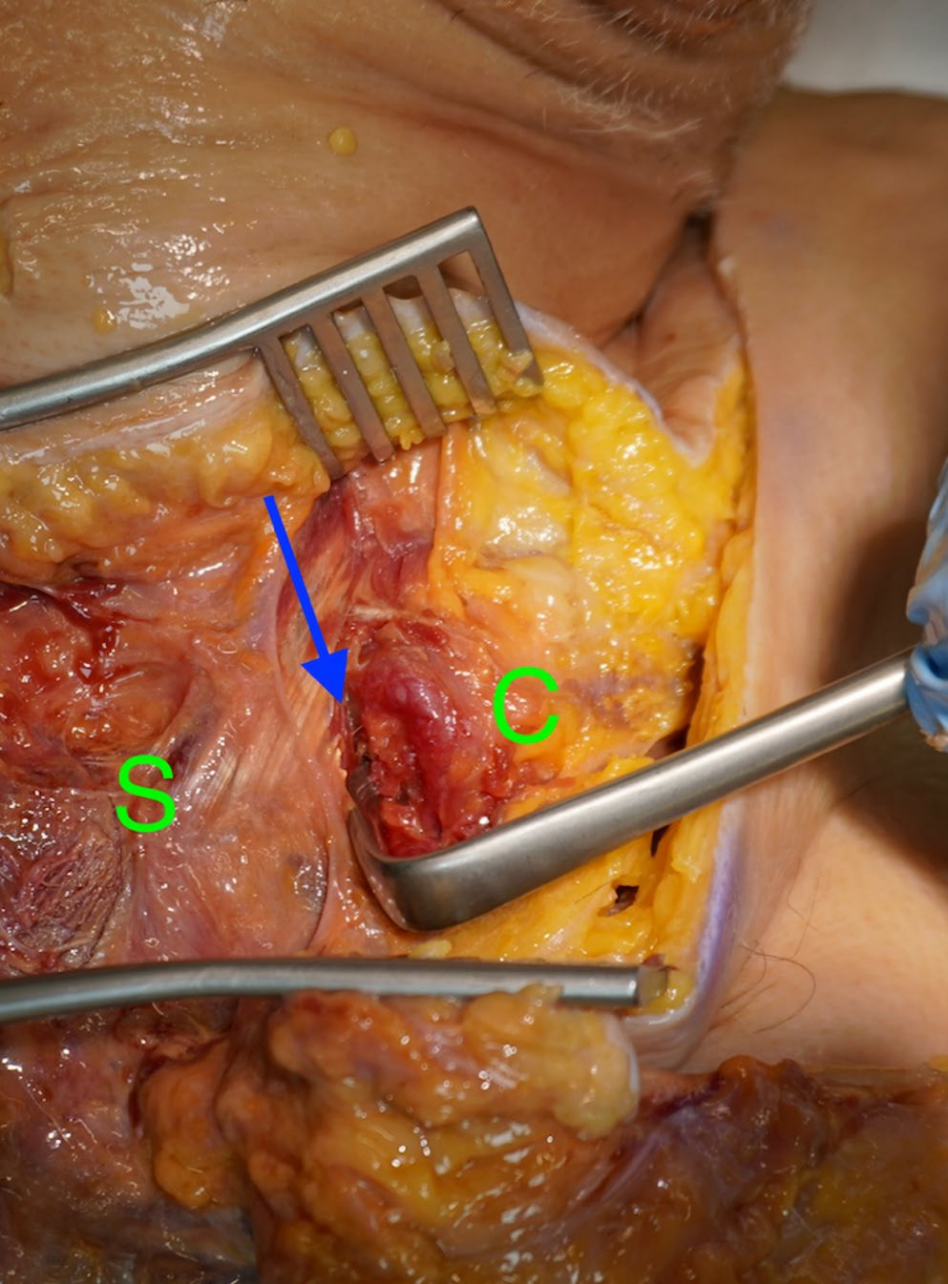
Anterior view of the SCJ (blue arrow). The sternum (green S) is visible medially, and the clavicle (green C) laterally. The ligaments were artificially transected to simulate injury and allow for surgical reconstruction

**Fig. 2:**
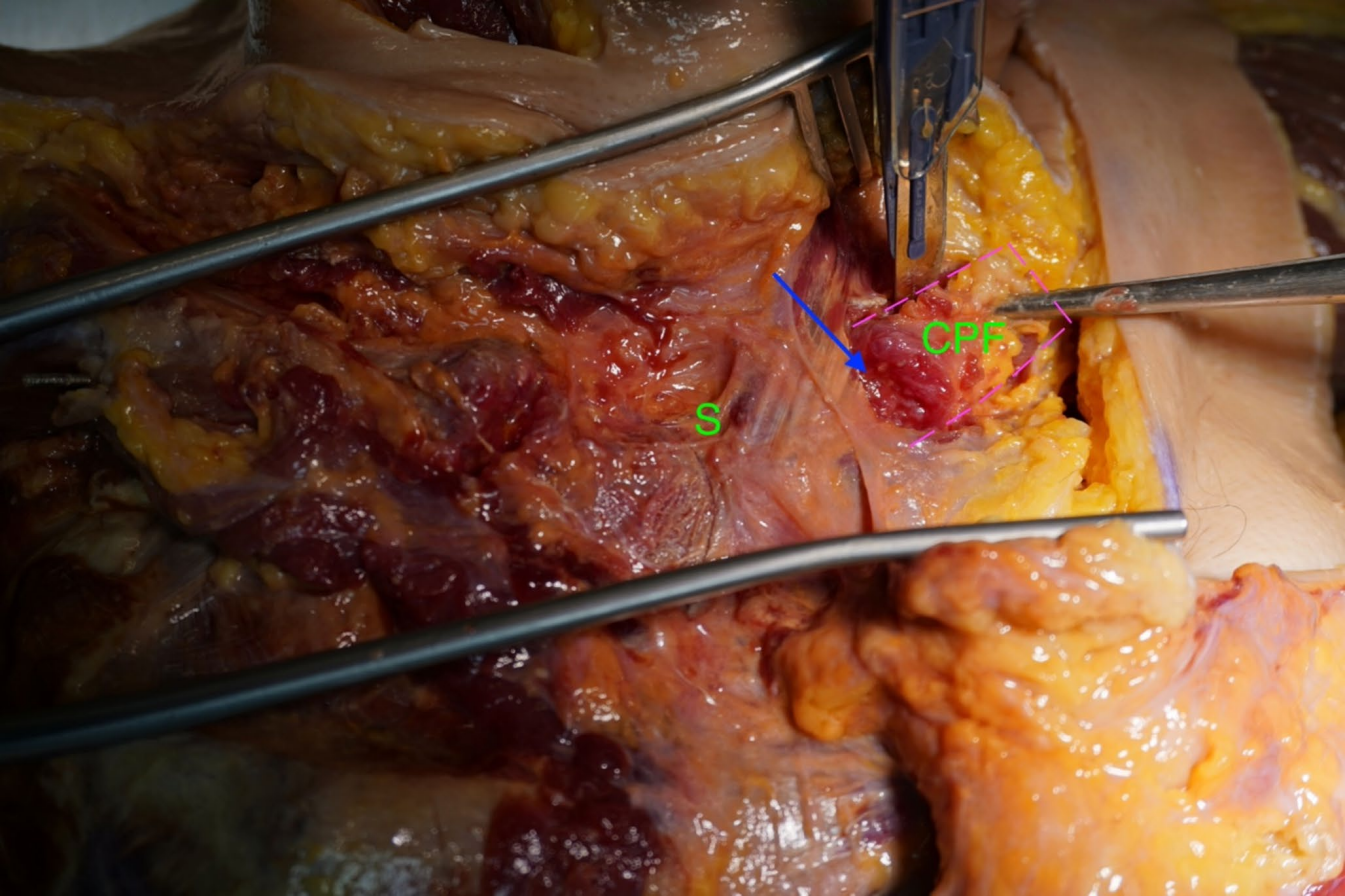
Anterior view of the SCJ (blue arrow) with preparation of the CPF from the clavicle. The sternum (green S) is shown centrally. The outline of the incision for preparation of the CPF is indicated by the pink dotted line

**Fig. 3:**
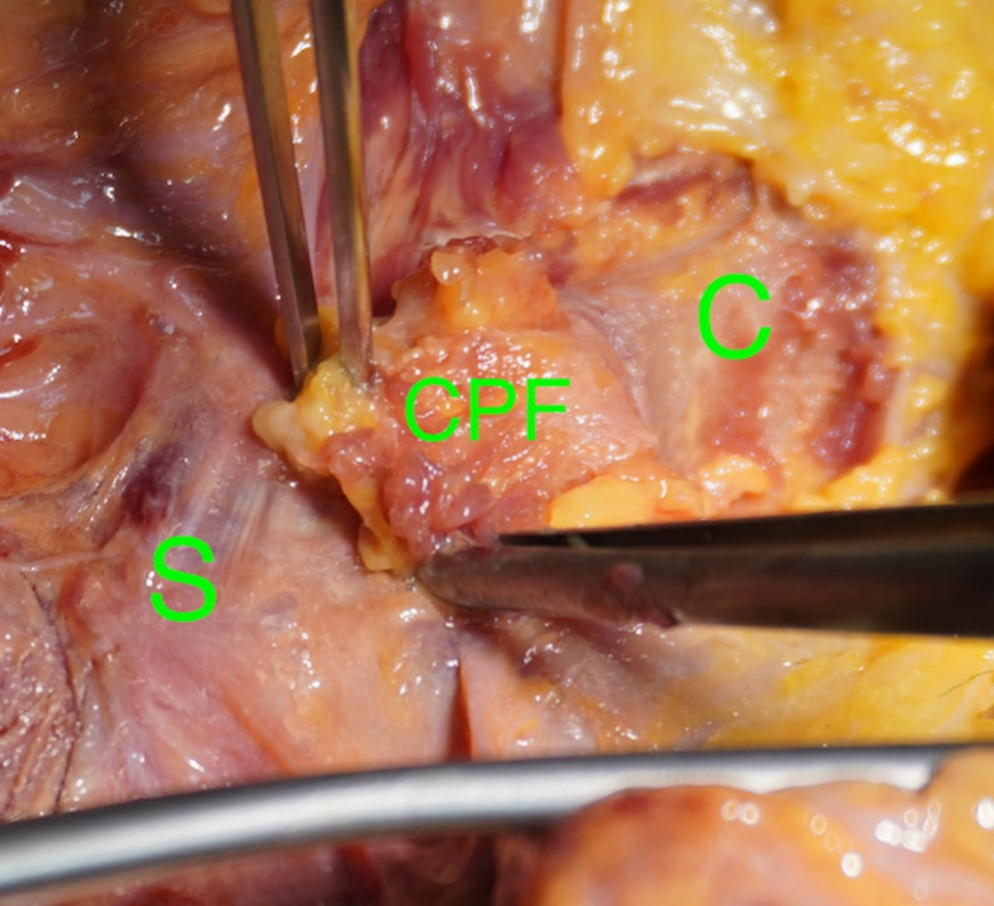
Anterior view after preparation of the CPF from the clavicle (green C). The CPF has been folded over the SCJ onto the sternum (green S)

**Fig. 4:**
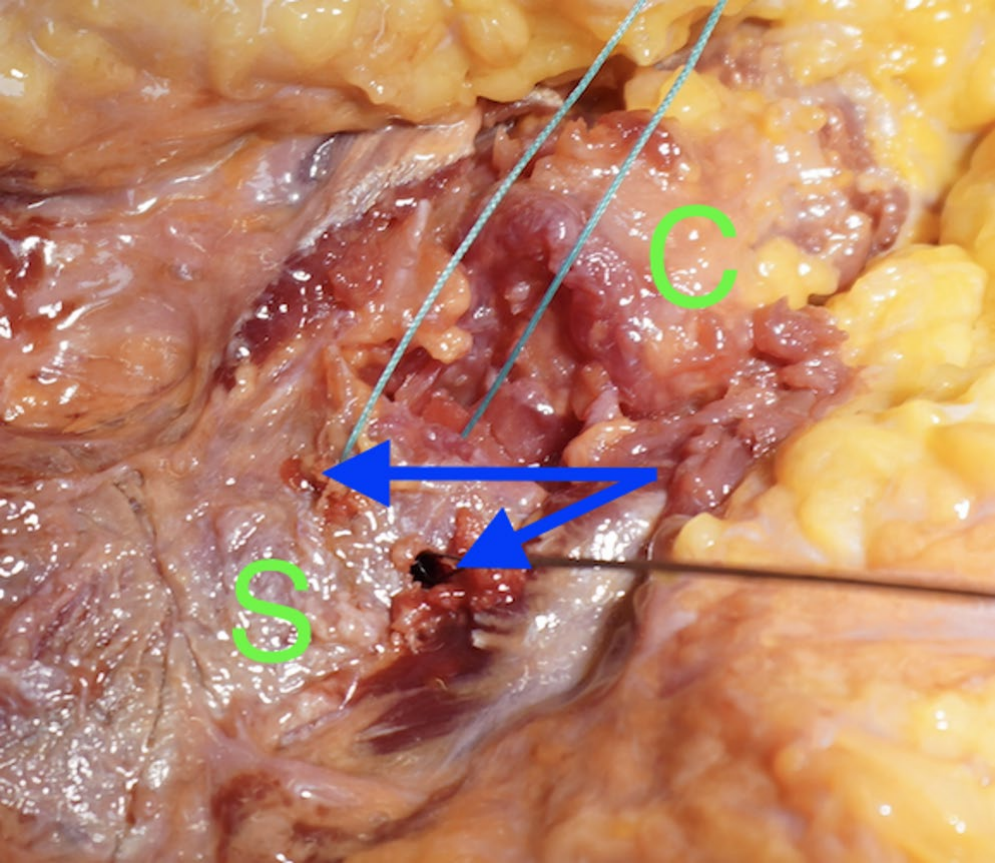
Anterior view of the SCJ. In this step, two 2-mm drill holes (blue arrows) were made through the sternum (green S). Using a shuttling device, the suture for later CPF fixation is passed through the holes. The clavicle (green C) is seen laterally

**Fig. 5:**
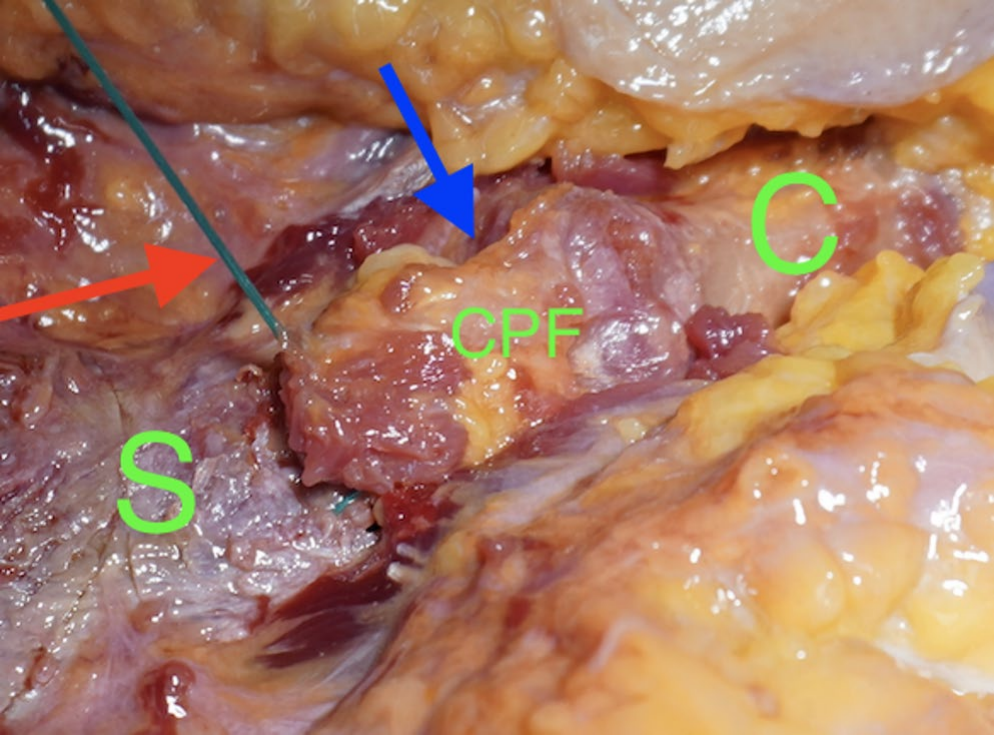
Anterior view of the final result. The CPF has been folded from the clavicle (green C) over the SCJ onto the sternum (green S) and secured with the suture (red arrow)

**Fig. 6:**
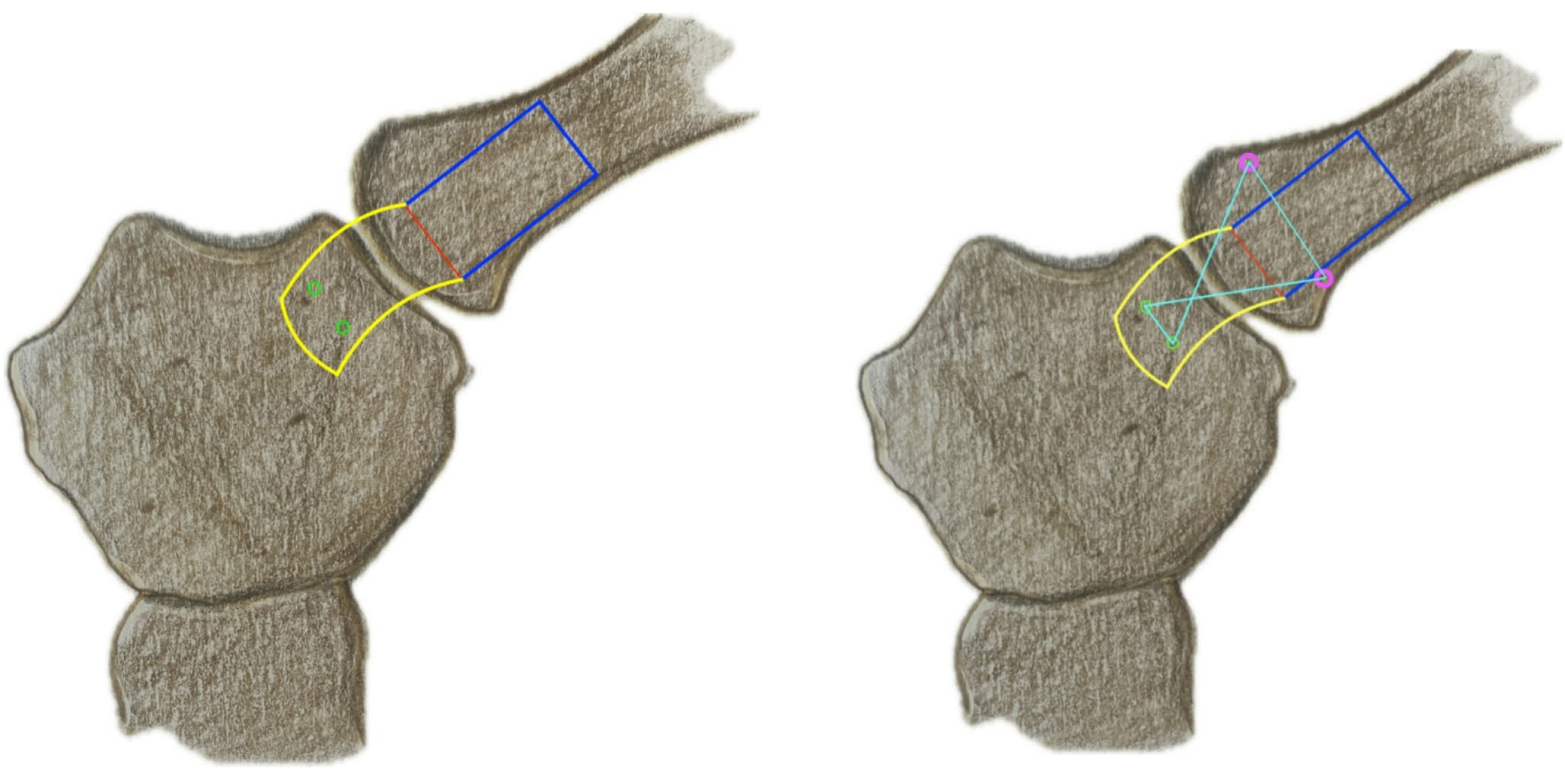
Schematic of the SCJ. Left: The clavicular periosteal flap (CPF) is mobilized from the blue-marked area of the clavicle while remaining attached at the red base. The yellow flap is then folded over the SCJ and fixed to the sternum via sutures passed through two green drill holes. Right: Additional clavicular drill holes (pink) are used to combine the CPF with figure-of-8 stabilization using a FiberWire (light blue)

We used a direct approach to the SCJ, employing a slightly larger incision to ensure optimal visualization of the manubrium sterni and the medial clavicle. The sternoclavicular joint was transected, as shown in Fig. 1. The CPF was mobilized from three sides, leaving the side closest and parallel to the SCJ intact. During preparation, care was taken not to detach the sternal origin of the sternocleidomastoid muscle or the costoclavicular ligament. The preparation of the CPF is shown in Fig. 2. Because the CPF was elevated on three sides and remained attached to the medial clavicle, large clavicular drill holes—such as those required for the figure-of-8 technique using a hamstring graft—could be avoided, allowing the CPF to be folded over the SCJ onto the sternum, as shown in Fig. 3. To assess the CPF, its length, width, and the extent of sternal overlap were measured. The sternal overlap was defined as the portion of the CPF that, after being folded over the SCJ, rested on the manubrium sterni. The free end of the CPF was reinforced using 2.0 FiberWire (Fa. Arthrex, Naples, FL, USA). Two sternal drill holes were created using a 1.6 mm drill. A rounded Hohmann retractor was placed behind the corresponding portion of the sternum to protect the retrosternal structures during drilling. Notably, the 1.6 mm drill holes were smaller than the 4 mm tunnels typically required for hamstring graft reconstructions. Instead of transsternal bicortical drill holes, unicortical drilling—as described by Imam et al.—could also be performed to minimize the risk of potential complications from injury to retrosternal structures [15]. The respective ends of the FiberWire were passed through the sternal drill holes using a shuttling device, positioning the CPF onto the manubrium sterni, where the sutures were tied above the CPF, as shown in Figs. 4 and 5. Afterwards, the FiberWire was passed through the 1.6 mm clavicular drill hole in a figure-of-8 fashion, as commonly performed in the acute setting. A schematic illustration of the surgical technique of the CPF can be seen in Fig. 6.

**Fig. 7:**
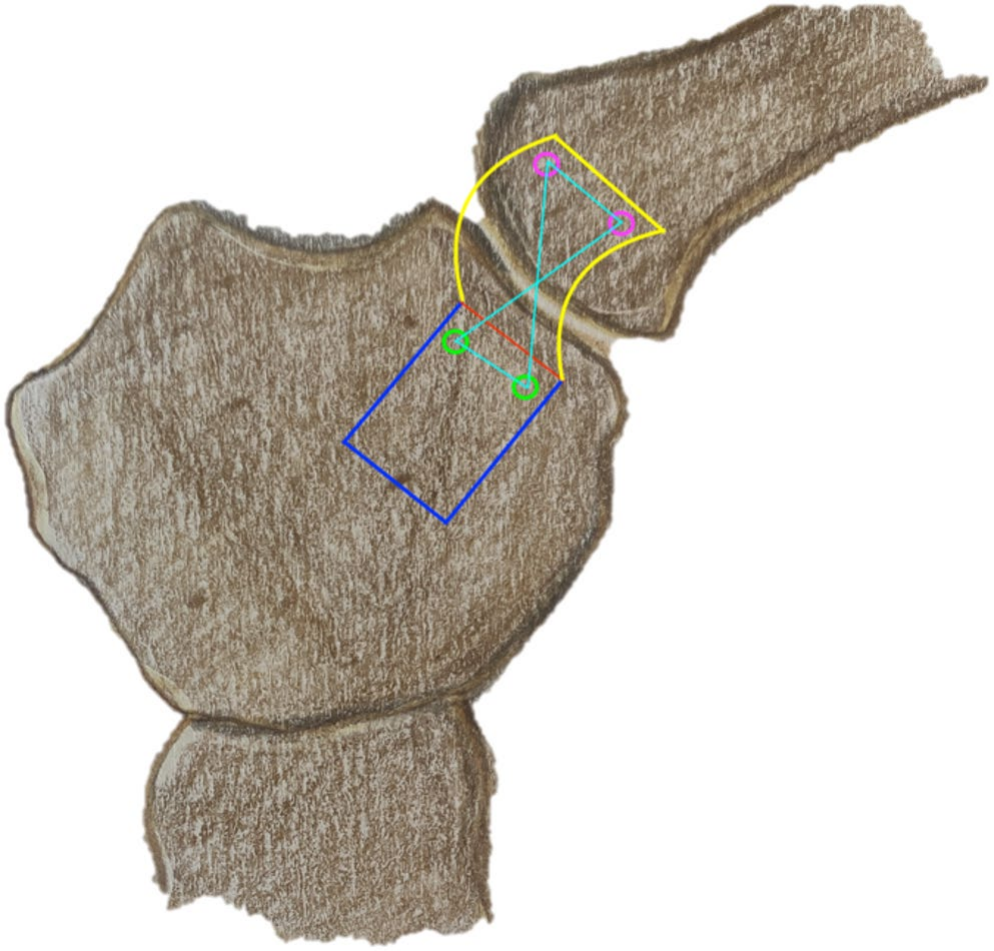
Schematic of the SCJ. The sternal periosteal flap (SPF) is mobilized from the blue-marked area of the sternum while remaining attached at the red base. The yellow flap is then folded over the SCJ and fixed to the clavicle via sutures passed through two pink drill holes. Additional sternal drill holes (green) are used to combine the SPF with a figure-of-8 stabilization using a FiberWire (light blue)

In the case of the SPF, the flap was detached from three sides of the sternum, preserving its attachment closest to the SCJ. It was then folded over the SCJ and fixed with a FiberWire passed through the 1.6 mm clavicular drill hole in a similar manner. The wire was subsequently passed through the sternum again to create a figure-of-8 brace. A schematic illustration of the surgical technique of the SPF can be seen in Fig. 7.

### Biomechanical testing

The CPFs were biomechanically tested using a uniaxial tensile testing machine (TH2730, Grip-Engineering, Nuremberg, Germany, software THSSD) to obtain values for stress at 50% strain (σ (50%)), maximum force (Fmax), maximum stress (σmax), strain at maximum force (ε(Fmax)), and strain at failure (ε at failure). For biomechanical testing, the periosteal flaps were detached at their SCJ-facing side, corresponding to the originally attached base. The flaps were not trimmed but exhibited comparable dimensions within a defined gauge length of 10 mm; their cross-sectional areas were individually measured optically. All samples were clamped using screw-action grips with rubber-coated steel jaws (type T240g+BV2, Fa. Grip-Engineering Thümler GmbH, Nuremberg, Germany) to prevent slippage. The clamps are suitable for tensile forces up to 5kN with a maximum clamping force of 10kN. All true load-to-failure tests were carried out with a preload force of 2 N and a pulling speed of 60 mm per minute. The maximum travel distance was limited to 80 mm and the maximum tensile force up to 60 N, thus significantly higher than the elongation break.

### Statistical analysis

Only descriptive statistics were applied. Continous variables are reported as means, standard deviations, and ranges. Bilateral joints obtained from the same cadaver were analyzed as individual measurements.

## Results

Mobilization of the prepared clavicular flaps was successfully achieved in all nine examined sternoclavicular joints. Clinical evaluation by the surgeons showed that the flaps were stable and tear-resistant, highlighting their potential suitability for surgical application. The prepared clavicular flaps had an average length of 3.5 ± 0.7 cm (range: 2.0–4.0 cm), a width of 2.6 ± 0.6 cm (range: 1.2–3.0 cm), and a sternal overlap of 2.0 ± 0.5 cm (range: 1.3–3.0 cm).

After preparation and fixation of the clavicular flaps, an additional attempt was made in four specimens to mobilize a sternal periosteal flap from the manubrium. This was also successfully achieved, with the sternal flaps showing an average length of 3.0 ± 0.05 cm (range: 2.9–3.0 cm), a width of 2.6 ± 0.5 cm (range: 2.0–3.0 cm), and an overlap onto the clavicle of 2.1 ± 0.1 cm (range: 2.0-2.2).

**Fig. 8:**
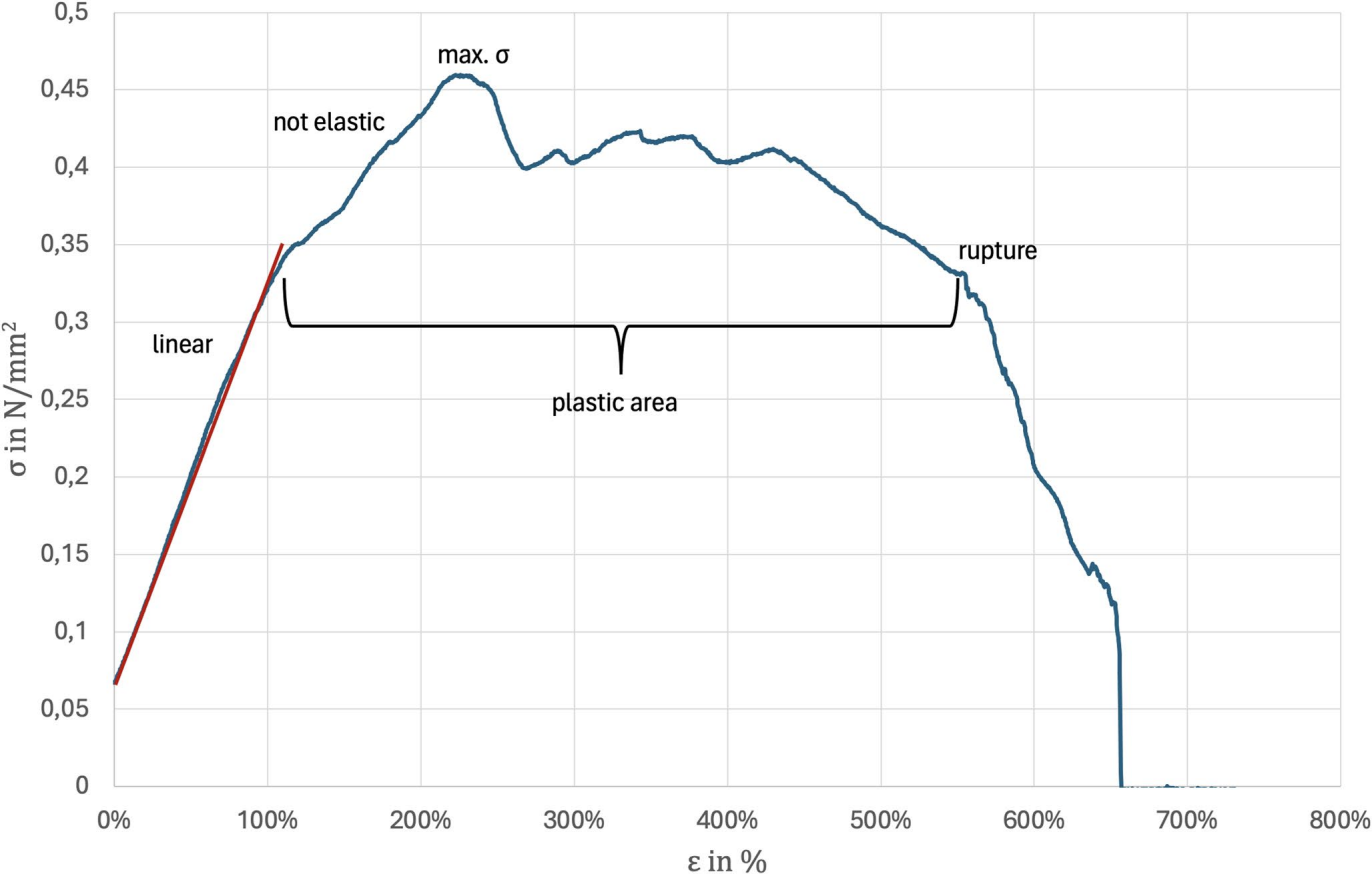
shows a representative stress-strain curve. The maximum tensile stress can be read at the highest point of the curve; beyond this point, the specimen begins to undergo necking. The endpoint of the curve represents the elongation at break, reflecting the maximum strain the specimen can sustain before failure. Failure was defined as either the spontaneous termination of the stress-strain curve or the first drop below the maximum elastic range

Biomechanical testing of the CPFs yielded a mean stress at 50% strain (σ (50%)) of 0.30 ± 0.12 N/mm^2^, a maximum force (Fmax) of 18.55 ± 9.0 N, a maximum stress (σmax) of 0.48 ± 0.10 N/mm^2^, a strain at maximum force (ε(Fmax)) of 1.69 ± 0.82%, and a strain at failure (ε at failure) of 2.36 ± 1.41%. Figure 8 shows a representative stress-strain curve. The maximum tensile stress can be read at the highest point of the curve; beyond this point, the specimen begins to undergo necking. The endpoint of the curve represents the elongation at break, reflecting the maximum strain the specimen can sustain before failure. Failure was defined as either the spontaneous termination of the stress-strain curve or the first drop below the maximum elastic range.

## Discussion

Various techniques have been described for the treatment of SCJ dislocations. In cases of acute traumatic dislocations, an initial trial of conservative management following successful reduction may be considered. Nevertheless, the rate of re-dislocation, particularly in ventral dislocations, remains relatively high [16, 17]. Despite this, many patients still achieve satisfactory and largely pain-free shoulder function. In cases of persistent symptoms — whether following acute trauma or due to chronic instability associated with hyperlaxity — surgical stabilization is indicated.

For both anterior and posterior dislocations, a variety of surgical methods have been reported, including stabilization using suture anchors, plating systems, and graft augmentation [18–20]. In recent years, figure-of-8 reconstruction using a hamstring graft has emerged as a preferred technique for SCJ stabilization. Favorable mid- and long-term outcomes, including 5- and 10-year follow-up results, have been demonstrated by Lacheta and Hinz et al. [9, 10] These studies showed that sternoclavicular SCJ reconstruction using a figure-of-8 hamstring tendon autograft led to significant improvements in shoulder function, pain reduction, high patient satisfaction, and a 90% construct survivorship at midterm (5-year) follow-up [10]. 

At minimum 10-year follow-up, these positive outcomes were maintained, with excellent function, low pain levels, and a 100% return-to-sport rate, although a 36% rate of recurrent subjective instability was noted [9]. Despite these promising results, there are some drawbacks to stabilization using a hamstring graft. Two clavicular and two sternal drill holes are required to tension the graft across the SCJ in a Fig. 8 configuration. These drill holes are typically created with a minimum of 4 mm diameter [10, 21, 22]. A study by Qui et al. demonstrated that the mean thickness in the coronal plane at the sternal end of the clavicle is 20.8 ± 6.0 mm, meaning that approximately 38% of the sternal clavicular thickness in the coronal plane is perforated by the drill holes, with some variation depending on the patient [23]. The study by Petri et al. 2016 evaluated clinical outcomes after SCJ reconstruction using hamstring tendon autografts in 21 patients with SCJ instability, demonstrating significant improvements in function and pain scores, high patient satisfaction, and no intraoperative or postoperative complications. Although good clinical outcomes were achieved in three patients, insufficient clavicular bone stock necessitated a modification from the standard figure-of-8 technique to a single drill hole with a loop reconstruction [24]. This finding indicates that insufficient clavicular bone stock is not uncommon, a limitation that would be obsolete with the technique demonstrated within the underlying article, as smaller clavicular drill holes are required only for the wire. In our cadaver study, the flaps were elevated on only three sides and remained attached near the SCJ. Additionally, the sternal and clavicular drill holes could be significantly reduced to 1.6 mm, which may help minimize the potential risk of fracture. Furthermore, it is possible to create two unicortical drill holes in the sternum that meet intraosseously, thereby minimizing the risk of injury to retrosternal structures [15]. A study by Tytherleigh-Strong et al. investigated a surgical approach for first-time traumatic anterior SCJ dislocations, involving direct repair of the anterior capsule augmented with an internal brace wire. Although no additional biological augmentation was performed, the technique aimed to restore stability in the acute setting by reinforcing the native anterior capsule. The reported outcomes were excellent, with no recurrent instability and a mean QuickDASH score of 2.3 at a median follow-up of 28 months [12]. Similar to their technique, we would also repair the capsule in the acute setting, followed by additional synthetic augmentation using the FiberWire and biological augmentation using the flap; in chronic cases with a torn and scarred capsule, the flap likewise offers the possibility of locally available biological augmentation. Another advantage of our technique is that the periosteal flaps, in contrast to tendon grafts, are highly vascularized and therefore provide superior healing potential. Furthermore, stabilization with a periosteal flap is much less prominent compared to the conventional figure-of-8 hamstring graft, which adds another benefit to our approach. Biomechanical testing showed that the CPF can withstand tensile forces on its own; however, as outlined in the surgical technique, primary stability is provided by the FiberWire fixation, while the locally available flap may support healing.

A limitation of our CPF technique could be, that not enough periosteal tissue is present at the clavicle or the sternum. In rare cases of particularly posterior dislocations, the authors observed within the surgery of clinical cases, that the periosteum may be sheared off together with the joint capsule. In those cases, the SPF could be an alternative.

Another advantage of our technique is that no hamstring graft harvesting from the knee is necessary. This avoids potential complications such as failed harvesting with premature tendon rupture or sensory deficits caused by injury to the infrapatellar branches of the saphenous nerve.

As early as 2006, Almazán et al. demonstrated that complications related to hamstring graft harvesting occur in 8.3% of cases. Furthermore, various studies have reported sensory deficits due to injury of the infrapatellar branches of the saphenous nerve in up to 88% of cases [25–27]. 

With our surgical technique, utilizing mobilization and fixation of a CPF or SPF, these potential complications can be avoided. While autografts may offer advantages in terms of biological incorporation and clinical outcomes compared to allografts, they are associated with the above-mentioned complications; therefore, alternative approaches such as locally available periosteal flaps warrant further investigations as potential biological augmentation [28]. 

This study has several limitations that should be considered when interpreting the results. First, the sample size was small, which is inherent to cadaveric feasibility investigations and limits the generalizability of the findings. Second, the body donors had a relatively advanced mean age, which represents a limitation, as patients with SCJ injuries are typically younger; however, it also demonstrates that the flaps can be mobilized even in older individuals despite age-related changes in the periosteum, including reduced thickness, cellularity, and regenerative capacity [29, 30].

Furthermore, biomechanical testing was only performed on the CPFs and was limited to the structural properties of the harvested flap tissue itself. The complete reconstruction construct, including the flap in combination with the figure-of-8 FiberWire augmentation, was not subject to construct-level biomechanical testing. Therefore, conclusions regarding the overall mechanical stability of the final reconstruction cannot yet be drawn.

Finally, this investigation represents a purely cadaveric anatomic feasibility study. Although cadaveric experiments allow standardized assessment of anatomy and tissue handling, they cannot reproduce biological healing, remodeling, or clinical outcomes.

Clinical studies are needed to validate this technique in both acute and chronic SCJ dislocations in the future.

## Conclusion

This cadaveric study provides an early proof-of-concept demonstrating the feasibility of a novel SCJ stabilization technique using a locally available CPF or SPF. While this approach may offer advantages by minimizing donor-site morbidity associated with graft harvesting and potentially reducing surgical complexity, further biomechanical and clinical studies are required to establish its effectiveness and clinical applicability.

## Data Availability

No datasets were generated or analysed during the current study.

## Abbreviations

SCJ: Sternoclavicular joint
CPF: Clavicular periosteal flap
SPF: Sternal periosteal flap
